# Is Aspirin Still Indispensable After PCI—Rethinking Dual Antiplatelet Therapy in Contemporary Practice

**DOI:** 10.3390/jcdd13050201

**Published:** 2026-05-09

**Authors:** Kartik Yadav, Sama Ehab Salah Ahmed, Mohamed Abdelgader, Roann Khalid, Murugapathy Veerasamy, Arka Das, Heerajnarain Bulluck

**Affiliations:** 1 Department of Cardiology, Leeds Teaching Hospitals NHS Trust, Leeds LS1 3EX, UK; kartik.yadav@nhs.net (K.Y.); sama.ahmed2@nhs.net (S.E.S.A.);; 2 General Medicine, King’s College Hospital NHS Foundation Trust, London SE5 9RS, UK; 3 Leeds Institute of Cardiovascular and Metabolic Medicine, University of Leeds, Leeds LS2 9JT, UK

**Keywords:** aspirin, percutaneous coronary intervention, dual antiplatelet therapy, P2Y12 inhibitor, ticagrelor, bleeding, stent thrombosis, de-escalation

## Abstract

Aspirin has been the default backbone of antiplatelet therapy after percutaneous coronary intervention (PCI) for over two decades, anchored by landmark trials that established 12-month dual antiplatelet therapy (DAPT) as the standard of care. Three developments have prompted reassessment of this paradigm: the markedly lower thrombotic risk of contemporary drug-eluting stents, the greater potency and consistency of potent P2Y_12_ inhibitors (ticagrelor, prasugrel), and increasing recognition that major bleeding independently worsens outcomes after PCI. Recent randomised trials have systematically tested aspirin withdrawal at varying time points. Immediate aspirin-free strategies (NEO-MINDSET, STOPDAPT-3) demonstrated an early signal of excess ischaemic events in the ACS component of enrolled populations, suggesting that aspirin remains important during the earliest post-PCI period in ACS. One-month strategies (T-PASS, ULTIMATE-DAPT, TARGET-FIRST) and three-month strategies (TWILIGHT, TICO, DUAL-ACS) showed that transition to P2Y_12_ monotherapy after an initial DAPT period significantly reduces bleeding without increasing ischaemic events in selected populations. Beyond one year, long-term randomised trials including the HOST-EXAM 10-year follow-up (Lancet 2026) and the STOPDAPT-2 5-year landmark analysis (Circ Cardiovasc Interv 2026), together with study-level meta-analyses (PANTHER) and recent individual patient data meta-analyses, provide converging evidence that clopidogrel monotherapy outperforms aspirin for chronic secondary prevention without excess bleeding. The choice of P2Y_12_ agent is critical: clopidogrel monotherapy in ACS during the first post-procedural year carries excess thrombotic risk owing to CYP2C19 pharmacogenomic variability, whereas ticagrelor and prasugrel provide more reliable protection. This review synthesises the mechanistic rationale, trial evidence across all time points, special clinical contexts (oral anticoagulation, coronary artery bypass grafting, high bleeding risk), guideline evolution, and methodological considerations, providing a practical framework for individualising post-PCI antiplatelet therapy.

## 1. Introduction

For over two decades, dual antiplatelet therapy (DAPT) with aspirin and a P2Y_12_ receptor inhibitor has been the default antithrombotic strategy after percutaneous coronary intervention (PCI) [1]. This paradigm was established by PCI-CURE, which showed that adding clopidogrel to aspirin reduced cardiovascular death, myocardial infarction (MI), and urgent revascularisation in non-ST-elevation acute coronary syndromes (ACS) [2], and reinforced by CREDO, in which 12 months of combined therapy reduced the composite of death, MI, or stroke by 26.9% compared with early clopidogrel discontinuation [3]. The Dual Antiplatelet Therapy (DAPT) Trial further extended this framework: continued therapy beyond 12 months reduced stent thrombosis and MI, but at the cost of increased bleeding and a troubling signal of higher non-cardiovascular mortality [4,5]. Twelve-month DAPT became the unchallenged default [6,7].

Three developments have since prompted reassessment of this paradigm. Second-generation drug-eluting stents (DES) with thinner struts and biocompatible polymers have markedly reduced late stent thrombosis, diminishing the absolute ischaemic risk that prolonged DAPT was originally designed to address [7,8,9]. Potent P2Y_12_ inhibitors—ticagrelor and prasugrel—provide more consistent and complete ADP-pathway blockade than clopidogrel, with superior ischaemic outcomes in large ACS trials [10,11]. Major bleeding after PCI is now recognised as an independent predictor of short- and long-term mortality, not merely an inconvenience [12].

The question is therefore not whether aspirin works, but whether it remains indispensable in an era of highly biocompatible stents and potent P2Y_12_ inhibition—and if not, in whom and at what point after PCI can it safely be withdrawn?

Recent randomised trials have systematically tested aspirin withdrawal and clopidogrel-versus-aspirin monotherapy at varying time points: immediately at PCI (NEO-MINDSET, STOPDAPT-3), after one month (T-PASS, ULTIMATE-DAPT, TARGET-FIRST), after three months (TWILIGHT, TICO, DUAL-ACS), and beyond one year (HOST-EXAM with 10-year follow-up published in The Lancet in 2026, the STOPDAPT-2 5-year landmark analysis published in Circulation: Cardiovascular Interventions in 2026, and PANTHER). The results depend critically on timing, clinical presentation, and the P2Y_12_ agent employed—three variables that define the organising framework of this review. Guideline bodies have begun to incorporate this evidence, recommending individualised DAPT duration based on validated risk stratification tools, including the ARC-HBR (Academic Research Consortium for High Bleeding Risk) criteria, rather than a uniform 12-month default [7,13].

This narrative review synthesises the mechanistic rationale, trial evidence across all time points, special clinical contexts, and practical considerations for individualising post-PCI antiplatelet therapy. The intent is not to argue that aspirin is obsolete, but to articulate a time-dependent role: indispensable in the immediate post-procedural window, increasingly dispensable thereafter in event-free patients receiving potent P2Y_12_ inhibition. Acute coronary syndrome (ACS) and chronic coronary syndrome (CCS) populations are not interchangeable, and where the evidence applies disproportionately to one or the other this is signposted throughout. Eligible studies were identified by structured PubMed and ClinicalTrials.gov searches (2010–2026) using the terms aspirin, dual antiplatelet therapy, P2Y_12_ inhibitor, percutaneous coronary intervention, and de-escalation, supplemented by hand searches of major society congress proceedings and reference lists of pivotal trials and meta-analyses; trials supportive of, neutral toward, and against aspirin-free strategies are all represented.

## 2. Mechanistic Rationale

Two platelet activation pathways are central to post-PCI antiplatelet therapy. Aspirin irreversibly acetylates cyclooxygenase-1 (COX-1), suppressing thromboxane A_2_ (TXA_2_) production and thereby inhibiting TXA_2_-mediated platelet recruitment and vasoconstriction [14]. The ADP–P2Y_12_ pathway operates in parallel: ADP released from activated platelets and damaged endothelium sustains and amplifies aggregation through P2Y_12_ receptor signalling, which is targeted by clopidogrel, prasugrel, and ticagrelor [15]. DAPT achieves dual-pathway blockade by simultaneously inhibiting both pathways.

The clinical relevance of this biology lies in how the balance between thrombotic risk and bleeding risk evolves over time after PCI. In the immediate post-procedural period, endothelial disruption and exposed stent struts create a highly thrombogenic environment in which dual-pathway blockade is well justified [14,15]. As stent endothelialisation progresses over weeks and months, device-related thrombogenicity declines, although cumulative aspirin exposure increases the risk of gastrointestinal and mucosal bleeding. The two pathways are not fully independent: TXA_2_-mediated activation requires ADP co-stimulation to produce stable thrombi under arterial shear, and conversely, ADP-driven aggregation is potentiated by TXA_2_. Consequently, the incremental benefit of dual blockade over monotherapy with a sufficiently potent P2Y_12_ agent may be smaller than previously assumed, particularly in the context of contemporary DES and event-free early recovery after PCI.

### Potent P2Y_12_ Inhibition and the Diminishing Incremental Value of Aspirin

The advent of potent P2Y_12_ inhibitors has materially altered this calculus. Clopidogrel exhibits substantial pharmacodynamic variability owing to its dependence on hepatic bioactivation via CYP2C19; loss-of-function polymorphisms, carried by 25–30% of Europeans and up to 60% of East Asians, reduce clopidogrel activation and are associated with excess ischaemic events after PCI [16,17]. Ticagrelor and prasugrel provide more reliable P2Y_12_ blockade. In PLATO, ticagrelor reduced the composite endpoint of cardiovascular death, MI, or stroke compared with clopidogrel (9.8% vs. 11.7%; HR 0.84, 95% CI 0.77–0.92), with a cardiovascular mortality benefit; ticagrelor did not increase overall PLATO-defined major bleeding but did significantly increase non-CABG-related major bleeding (4.5% vs. 3.8%; *p* = 0.03) and fatal intracranial bleeding [10]. TRITON–TIMI 38 showed prasugrel reduced the composite ischaemic endpoint (9.9% vs. 12.1%; HR 0.81, 95% CI 0.73–0.90) at the cost of increased TIMI major bleeding, including fatal bleeding [11]. Both agents therefore deliver superior ischaemic protection over clopidogrel, but at the price of an increment in spontaneous, non-procedural bleeding events.

**Figure 1:**
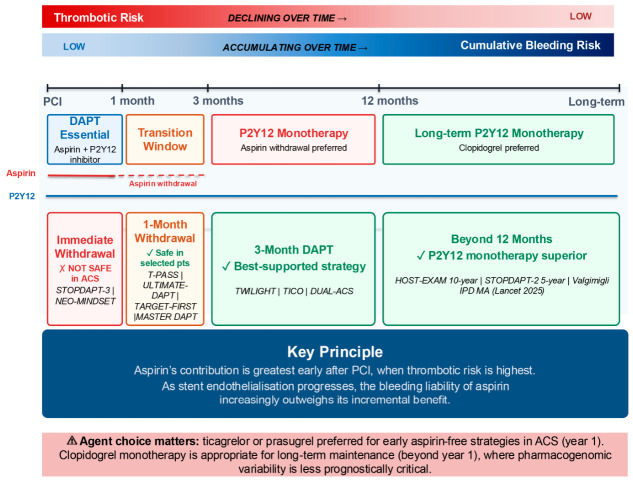
The Time-Dependent Role of Aspirin after PCI.

These trials established that ADP-mediated platelet activation is a dominant driver of post-PCI thrombotic risk, and that potent, consistent P2Y_12_ blockade delivers the majority of antithrombotic benefit. When P2Y_12_ receptor blockade is near-complete, residual TXA_2_-mediated platelet activation may be insufficient on its own to sustain clinically relevant thrombus formation [10,11,14]. This does not imply that aspirin provides no benefit; rather, its incremental contribution over potent P2Y_12_ monotherapy may be small enough in certain patients and at certain time points to be outweighed by its bleeding liability. The evolving balance between declining thrombotic risk and accumulating bleeding liability after PCI is summarised in Figure 1.

When evaluating the trial evidence that follows, three considerations warrant attention: the potency and reliability of the P2Y_12_ agent used; the thrombogenic risk profile of the population (ACS vs. chronic coronary syndrome; complex vs. simple PCI); and the timing of aspirin withdrawal relative to the highest-risk peri-procedural period.

## 3. Immediate Aspirin Withdrawal at PCI

Immediate or ultra-early aspirin-free strategies represent the most aggressive approach to aspirin sparing after PCI. The rationale is straightforward: if potent P2Y_12_ inhibition provides the dominant antithrombotic effect, aspirin could theoretically be omitted from the outset, eliminating its bleeding contribution entirely. The problem is that this strategy removes aspirin during the phase of highest thrombotic vulnerability after ACS, when plaque rupture, tissue factor exposure, and heightened platelet–monocyte interactions create a profoundly prothrombotic milieu [18,19]. Patients with ACS have greater thrombin generation capacity, higher factor XIa levels, and markedly higher spontaneous platelet activation than those with stable coronary disease, with ST-elevation MI (STEMI) displaying the most prothrombotic profile [20].

STOPDAPT-3 was the first large randomised trial to test whether aspirin could be stopped immediately at the time of PCI [21]. It enrolled 5966 patients with ACS or high bleeding risk just before PCI; 75% had ACS. Patients received either prasugrel monotherapy at the Japan-approved dose of 3.75 mg or DAPT with aspirin plus prasugrel. At one month, prasugrel monotherapy was not superior for major bleeding (BARC 3/5: 4.47% vs. 4.71%; HR 0.95, 95% CI 0.75–1.20), although it met non-inferiority for the cardiovascular composite (4.12% vs. 3.69%; HR 1.12, 95% CI 0.87–1.45) [21]. That overall non-inferiority result requires careful interpretation, because the aspirin-free strategy was associated with more unplanned coronary revascularisation (1.05% vs. 0.57%; HR 1.83) and significantly more early (within 30 days) definite or probable stent thrombosis (0.58% vs. 0.17%; HR 3.40, 95% CI 1.26–9.23) [21]. In the high-bleeding-risk ACS subgroup, aspirin withdrawal still failed to reduce major bleeding, whilst MI was more frequent with the aspirin-free approach (1.6% vs. 0.3%; HR 4.57) [22]. These findings are consistent with the underlying biology: in ACS, especially early after PCI, reducing antiplatelet intensity exposes a real thrombotic penalty before any bleeding advantage becomes evident.

NEO-MINDSET tested the same concept in a pure ACS population using contemporary potent P2Y_12_ inhibitors [23]. In 3410 patients undergoing successful PCI for ACS, aspirin was withdrawn within four days of hospitalisation in the monotherapy arm. Potent P2Y_12_ monotherapy failed to achieve non-inferiority for the primary ischaemic composite of death, MI, stroke, or urgent revascularisation at 12 months (7.0% vs. 5.5%; absolute risk difference 1.47 percentage points; 95% CI −0.16–3.10; *p* = 0.11 for non-inferiority), although bleeding was substantially lower (2.0% vs. 4.9%) [23]. Stent thrombosis was numerically higher with monotherapy (12 vs. 4 events). The STEMI substudy further clarified the risk gradient: among STEMI patients, early aspirin withdrawal increased ischaemic events compared with DAPT (8.2% vs. 5.2%; HR 1.60, 95% CI 1.14–2.24), whereas in NSTE-ACS, ischaemic rates were similar [24]. This strongly suggests that the more thrombogenic the ACS phenotype, the less safe immediate aspirin withdrawal becomes.

Taken together, the available randomised evidence does not support routine immediate aspirin withdrawal at PCI in unselected ACS. Although bleeding was reduced with aspirin-free therapy in NEO-MINDSET, the accompanying increase in coronary events—particularly stent thrombosis and MI in STEMI—limits the clinical acceptability of immediate aspirin withdrawal. Aspirin remains indispensable during the first weeks after PCI for ACS.

## 4. Aspirin Withdrawal at One Month

The excess thrombotic events observed with immediate aspirin withdrawal raised the question of whether a brief initial period of DAPT—sufficient to cover the highest-risk peri-procedural window—might permit safe transition to P2Y_12_ monotherapy. Several trials have now tested this approach, with aspirin discontinued approximately two to four weeks after PCI.

### 4.1. Ticagrelor Monotherapy After Short DAPT

T-PASS randomised 2850 ACS patients in South Korea to aspirin discontinuation at a median of 16 days after PCI, with ticagrelor monotherapy thereafter, versus standard 12-month DAPT [25]. The primary composite of net adverse clinical events (NACE) was significantly lower with early aspirin withdrawal (2.8% vs. 5.2%; HR 0.54, 95% CI 0.37–0.80), driven by a halving of major bleeding with no excess MI or stent thrombosis. Approximately 40% of participants had STEMI. The population was moderately low-risk, and all patients were East Asian, which warrants caution when extrapolating to other ethnic groups.

ULTIMATE-DAPT enrolled approximately 3400 ACS patients who had completed one month of event-free DAPT, randomising them to ticagrelor plus placebo versus ticagrelor plus aspirin for months 1–12 [26]. Clinically relevant bleeding was significantly reduced (2.1% vs. 4.6%; HR 0.45), and the composite of major adverse cardiovascular and cerebrovascular events (MACCE) was 3.6% versus 3.7%—meeting non-inferiority [26]. The trial demonstrates that once the initial month of DAPT is safely navigated, the bleeding benefit of aspirin withdrawal with ticagrelor monotherapy is substantial without measurable ischaemic cost.

GLOBAL LEADERS adopted a different design, randomising 15,968 all-comers PCI patients—including a substantial proportion of CCS patients—to one month of ticagrelor plus aspirin followed by 23 months of ticagrelor monotherapy versus standard DAPT and then aspirin [27]. This design differs importantly from the ACS-focused one-month trials above: it enrolled a heterogeneous population and evaluated an extended monotherapy period rather than a discrete aspirin withdrawal strategy. The primary endpoint of all-cause death or new Q-wave MI at two years did not reach significance (rate ratio 0.87, 95% CI 0.75–1.01; *p* = 0.073). No early bleeding benefit was observed, though rates of BARC 3–5 bleeding were numerically lower with the experimental strategy. The heterogeneous all-comer population likely diluted any treatment effect. GLOBAL LEADERS demonstrated the feasibility of early aspirin withdrawal in a broad population, though it failed to establish superiority.

TARGET-FIRST randomised 1942 low-risk, completely revascularised acute MI patients who remained event-free after one month of DAPT to P2Y_12_ monotherapy versus continued DAPT for 11 months [28]. Ischaemic non-inferiority was met (absolute difference −0.09 percentage points), and BARC 2/3/5 bleeding was halved (2.6% vs. 5.6%; HR 0.46, 95% CI 0.28–0.76; *p* = 0.002) [28]. These data support the feasibility of early aspirin withdrawal in carefully selected low-risk MI patients who have achieved complete revascularisation.

### 4.2. Population and Timing Considerations

Overall, one-month strategies appear safer than immediate aspirin withdrawal, supporting the concept that dual-pathway inhibition is most critical during the earliest post-PCI period. Pooled individual patient data from TICO and T-PASS confirm consistent bleeding reduction with ticagrelor monotherapy after short DAPT, with preserved ischaemic outcomes [29].

Several considerations temper enthusiasm for routine one-month aspirin withdrawal. T-PASS, ULTIMATE-DAPT, and TARGET-FIRST were conducted predominantly in East Asian or selected European cohorts; wider ethnic representativeness is needed before these strategies can be considered standard. The P2Y_12_ agent matters: these trials used ticagrelor (or investigator-choice potent agents), not clopidogrel—a distinction that becomes critical in the context of pharmacogenomic variability discussed in Section 6. Finally, patient selection was stringent in most trials, focusing on event-free survivors at one month; applicability to the full spectrum of post-PCI patients, including those with complex interventions or ongoing ischaemic features, requires individualised assessment.

Taken together, one-month aspirin withdrawal with transition to potent P2Y_12_ monotherapy consistently reduces bleeding without measurable ischaemic cost in trials using ticagrelor in selected, event-free populations. The strategy is reasonable for lower-risk ACS patients who remain stable through the initial DAPT period, but cannot yet be generalised to all post-PCI patients or to clopidogrel-based regimens.

## 5. Three-Month DAPT Strategies

Among abbreviated strategies, three months of DAPT followed by P2Y_12_ inhibitor monotherapy is supported by the largest body of randomised evidence, including trials enrolling high-risk populations.

TWILIGHT remains the landmark trial in this space. It randomised 7119 high-risk PCI patients who had completed three months of event-free DAPT with ticagrelor plus aspirin to continue aspirin or switch to placebo, with ticagrelor continued in both arms [30]. BARC 2–5 bleeding was reduced by 44% with ticagrelor monotherapy (4.0% vs. 7.1%; HR 0.56, 95% CI 0.45–0.68; *p* < 0.001), with a number needed to treat of approximately 32. The composite of death, MI, or stroke was non-inferior (3.9% vs. 3.9%; HR 0.99) [30]. Subgroup analyses confirmed that these benefits were reproduced in patients meeting ARC-HBR criteria and in those with anaemia, populations at particularly high bleeding risk. The three-month run-in design, however, ensured that only event-free patients were randomised—a strength for internal validity but a limitation for generalisability to the full PCI population.

TICO enrolled approximately 3000 ACS patients undergoing PCI in Republic of Korea, randomising them to ticagrelor monotherapy after three months of DAPT versus standard 12-month DAPT [31]. NACE was significantly reduced with the abbreviated strategy, driven principally by lower bleeding; ischaemic outcomes were non-inferior [31]. The trial complemented TWILIGHT by demonstrating similar benefits in an exclusively ACS population, albeit predominantly East Asian.

DUAL-ACS, the largest pragmatic aspirin withdrawal trial to date, randomised more than 5000 MI patients across European centres to three months of DAPT followed by P2Y_12_ monotherapy versus standard 12-month DAPT [32]. In this pragmatic, open-label design, preliminary results suggested a trend towards lower all-cause mortality (HR 0.78) and major bleeding (HR 0.78) with abbreviated DAPT, with no increase in cardiovascular death or non-fatal MI [32]. Although neither outcome reached conventional statistical significance, the consistent direction of effect across both ischaemic and bleeding endpoints provides an encouraging signal from the largest pragmatic RCT to date testing three-month DAPT in the broad MI population. It should be noted that at the time of writing, DUAL-ACS results have been reported as a conference presentation pending peer-reviewed publication, and the findings described here should be interpreted accordingly.

MASTER-DAPT addressed the specific question of abbreviated DAPT in patients meeting ARC-HBR criteria. Approximately 4600 HBR patients (*n* = 4579) were randomised to one to three months of DAPT versus standard or prolonged therapy [33]. The abbreviated strategy was non-inferior for ischaemic outcomes, and clinically relevant bleeding was significantly lower (absolute difference −2.82 percentage points; *p* < 0.001) [33]. MASTER-DAPT did not mandate a specific monotherapy agent, reflecting real-world variability; most patients received clopidogrel, given the high prevalence of concomitant oral anticoagulation and advanced age in this population.

SMART-CHOICE and STOPDAPT-2 provide additional supporting data in less acute populations. SMART-CHOICE randomised approximately 3000 patients—predominantly CCS—to P2Y_12_ monotherapy (mainly clopidogrel) after three months of DAPT, demonstrating non-inferior ischaemic outcomes [34]. STOPDAPT-2 showed superior net clinical outcomes with one-month DAPT followed by clopidogrel monotherapy versus 12-month DAPT in a mixed CCS/ACS population, driven by reduced bleeding [35]. Both trials were conducted exclusively in East Asian populations, and STOPDAPT-2’s findings were subsequently tempered by the ACS subgroup analysis discussed in Section 6.

Taken together, these trials illustrate a consistent pattern: three months of DAPT followed by P2Y_12_ monotherapy significantly reduces bleeding without increasing ischaemic events across a range of post-PCI populations. The evidence supporting a three-month de-escalation is primarily derived from randomised trials (TWILIGHT, TICO, DUAL-ACS), and this time point appears to offer the most favourable trade-off between efficacy and safety for a broad ACS population. The choice of P2Y_12_ agent and the population characteristics remain important determinants of safety.

The key design features and outcomes of the pivotal randomised trials are summarised in Table 1.

## 6. The Clopidogrel Problem: Why Agent Choice Matters

Not all P2Y_12_ inhibitors behave equally when aspirin is withdrawn. Clopidogrel’s pharmacogenomic variability has clinically important implications, particularly when aspirin is removed, and antithrombotic protection depends entirely on P2Y_12_ inhibition.

STOPDAPT-2 demonstrated superior net clinical outcomes with one-month DAPT followed by clopidogrel monotherapy in a mixed population [35]. When the same strategy was tested specifically in ACS patients (STOPDAPT-2 ACS) at one year, clopidogrel monotherapy failed to achieve non-inferiority for the composite NACE endpoint (HR 1.14; *p* for non-inferiority = 0.06), with a numerical increase in cardiovascular events (2.8% vs. 1.9%) despite reduced bleeding [36]. The ischaemic protection provided by clopidogrel was therefore insufficient as a substitute for DAPT during the first post-procedural year in the higher-thrombotic-risk ACS setting. The 5-year landmark analysis, published in 2026, has refined this picture in an entirely different direction for chronic monotherapy [37]. From one year to five years, in the STOPDAPT-2 ACS cohort (*n* = 2986), clopidogrel was superior to aspirin for the primary composite endpoint (6.18% vs. 8.27%; HR 0.75, 95% CI 0.57–0.997; *p* = 0.048) and for the cardiovascular composite (4.73% vs. 6.77%; HR 0.70, 95% CI 0.51–0.96; *p* = 0.03). In the STOPDAPT-2 total cohort (*n* = 5991), the cardiovascular endpoint favoured clopidogrel (HR 0.74, 95% CI 0.60–0.91; *p* = 0.004) without a difference in TIMI major or minor bleeding. These data clarify the time-dependent picture: clopidogrel monotherapy is unsafe as a substitute for DAPT in the first year after ACS, but is the preferred chronic monotherapy thereafter for event-free patients.

The mechanistic explanation centres on CYP2C19 pharmacogenomic variability. Clopidogrel is a prodrug requiring hepatic bioactivation via CYP2C19 to generate its active metabolite. Loss-of-function (LOF) alleles, predominantly CYP2C19*2 and CYP2C19*3, are present in 25–30% of Europeans and up to 60% of East Asians [16]. Carriers of these alleles exhibit reduced clopidogrel activation, higher on-treatment platelet reactivity, and excess thrombotic events—particularly stent thrombosis—after PCI [16,17]. When aspirin is withdrawn, and the patient depends entirely on clopidogrel for antithrombotic protection, the consequences of subtherapeutic P2Y_12_ inhibition become clinically significant. This pharmacogenomic dependence is precisely what differentiates clopidogrel monotherapy from ticagrelor monotherapy after PCI. Ticagrelor, a direct-acting reversible P2Y_12_ antagonist, requires no hepatic bioactivation and is unaffected by CYP2C19 genotype, so its pharmacodynamic effect is uniform across populations. A pooled patient-level analysis of randomised trials of P2Y_12_ inhibitor monotherapy after short DAPT in ACS confirmed this clinically: ticagrelor monotherapy reduced ischaemic and bleeding events consistently across CYP2C19 strata [38]; clopidogrel monotherapy, by contrast, shows attenuated ischaemic protection in loss-of-function carriers [16,17]. In practice, when an aspirin-free regimen is contemplated in an ACS patient without genotype information, ticagrelor is the more pharmacogenomically robust choice; clopidogrel monotherapy is justifiable only when normal-metaboliser status has been confirmed or when the clinical phenotype favours a less potent agent (CCS, low ischaemic risk, established intolerance or contraindication to ticagrelor or prasugrel).

The STOPDAPT-3 findings corroborate this principle from a different angle: even low-dose prasugrel—a more potent but still metabolically activated agent—failed to prevent excess stent thrombosis when aspirin was withdrawn immediately at PCI [21]. This indicates that both timing and potency matter and that simply using a more potent P2Y_12_ inhibitor does not fully compensate for aspirin omission in the highest-risk period.

POPular Genetics provided the most rigorous evidence for genotype-guided antiplatelet selection [39]. In STEMI patients undergoing primary PCI, a CYP2C19 genotype-guided strategy—in which carriers of LOF alleles received ticagrelor or prasugrel and non-carriers were assigned to clopidogrel—was compared with standard treatment consisting of ticagrelor or prasugrel for 12 months, irrespective of genotype. The guided strategy was non-inferior for the combined thrombotic and bleeding composite (5.1% vs. 5.9%; absolute difference −0.7 percentage points; *p* < 0.001 for non-inferiority) and significantly reduced bleeding (9.8% vs. 12.5%; HR 0.78, 95% CI 0.61–0.98) [39]. This trial supports the precise use of clopidogrel in genotype-selected patients, though it evaluated genotype-guided selection within 12 months of DAPT rather than as monotherapy after abbreviated DAPT.

The practical implication is clear: ticagrelor and, to a lesser extent, prasugrel are the preferred P2Y_12_ agents for aspirin-free strategies in ACS. Clopidogrel monotherapy may be appropriate in CCS settings, in genotype-confirmed normal metabolisers, or in patients with contraindications to potent P2Y_12_ inhibitors, but it should not be the default choice when aspirin is withdrawn early after ACS-related PCI.

## 7. Tailored De-Escalation: Dose Reduction and Combination Strategies

Beyond agent switching and aspirin withdrawal, dose reduction and combination strategies offer further personalisation—particularly relevant for patients at simultaneous high bleeding and high ischaemic risk, in whom standard approaches may be insufficiently nuanced.

4D-ACS randomised 1370 ACS patients undergoing PCI with DES to one month of prasugrel-based DAPT followed by prasugrel 5 mg monotherapy versus standard 12-month DAPT [40]. NACE was significantly lower with the de-escalation strategy (4.9% vs. 8.8%), meeting both non-inferiority and superiority criteria, driven by a substantial reduction in BARC 2–5 bleeding (1.4% vs. 6.1%; HR 0.23, 95% CI 0.13–0.42), equating to a 77% relative risk reduction; ischaemic outcomes were non-inferior [40]. The trial is notable for combining early aspirin withdrawal with dose de-escalation of the P2Y_12_ agent, addressing both bleeding drivers simultaneously.

HOST-REDUCE-POLYTECH-ACS evaluated structured prasugrel dose de-escalation from 10 mg to 5 mg in ACS patients after PCI [41]. The de-escalated arm demonstrated significantly lower bleeding (HR 0.48, 95% CI 0.32–0.73) with non-inferior ischaemic protection, reinforcing the principle that maintenance-phase P2Y_12_ inhibitor dose can be reduced in appropriately selected patients without compromising safety.

OPT-BIRISK addressed the under-studied population of patients with both high bleeding and high ischaemic risk—a group for whom standard treatment algorithms often provide conflicting recommendations [42]. In 7758 ACS patients meeting both high-risk criteria, extended clopidogrel monotherapy for nine months after 12 months of DAPT was compared with continued DAPT. BARC 2, 3, or 5 bleeding was less frequent with clopidogrel (2.5% vs. 3.3%; HR 0.75, 95% CI 0.58–0.97; *p* = 0.03), and MACCE was also lower (2.6% vs. 3.5%; HR 0.74; *p* for non-inferiority < 0.001) [42]. These data suggest that extended P2Y_12_ inhibitor monotherapy may benefit patients at dual risk, a finding of direct relevance to the growing proportion of PCI patients who are elderly, have renal impairment, or carry multiple comorbidities.

TROPICAL-ACS tested platelet function-guided de-escalation from prasugrel to clopidogrel, using the Multiplate assay to identify patients with adequate clopidogrel response [43]. The guided strategy was non-inferior to the standard strategy for the primary ischaemic and bleeding composite at 12 months [43]. Although the trial evaluated DAPT de-escalation rather than aspirin withdrawal per se, it provides proof of concept that biology-guided antiplatelet personalisation is feasible and safe.

These tailored approaches illustrate that the optimal post-PCI strategy depends not only on whether aspirin is continued, but also on the choice and dose of the accompanying P2Y_12_ inhibitor. Combining early aspirin withdrawal with dose de-escalation, as in 4D-ACS, or extending P2Y_12_ monotherapy in dual-risk patients, as in OPT-BIRISK, represents a move towards truly individualised antiplatelet therapy—a framework rather than a single protocol.

## 8. Guideline Evolution and Risk Stratification

### 8.1. Current Guidelines

The 2023 ESC guidelines for ACS management recommend 12 months of DAPT with aspirin plus a potent P2Y_12_ inhibitor (ticagrelor or prasugrel) as the default strategy after PCI for ACS [6]. Abbreviated DAPT (one to three months) is endorsed as a Class IIa recommendation for patients at high bleeding risk, with subsequent transition to P2Y_12_ monotherapy. De-escalation from a potent P2Y_12_ inhibitor to clopidogrel is acknowledged as a Class IIb option. The guidelines emphasise that selection of DAPT duration should incorporate individual bleeding and ischaemic risk assessment rather than relying on a uniform default [6]. The 2024 ESC guidelines for the management of chronic coronary syndromes complement this framework on the CCS side, recommending an individualised DAPT duration after PCI based on the balance between ischaemic and bleeding risk, with shorter regimens favoured for patients meeting ARC-HBR criteria [44].

The 2025 ACC/AHA guidelines adopt a similarly flexible framework [45]. Twelve months of DAPT remains the default for most ACS patients not at high bleeding risk, but transition to ticagrelor monotherapy after at least one month of DAPT is endorsed as a high-class recommendation for patients with high bleeding risk [45]. De-escalation to clopidogrel or single antiplatelet therapy after one month carries a lower recommendation class. Both transatlantic guidelines converge on the principle that aspirin’s role after PCI is time-limited and risk-dependent, though neither has yet fully incorporated the most recent trial data from DUAL-ACS, TARGET-FIRST, and NEO-MINDSET.

### 8.2. Risk Stratification Tools

Effective implementation of individualised DAPT requires validated tools for assessing competing bleeding and ischaemic risks.

The PRECISE-DAPT score, derived from retrospective analysis of eight randomised trials and externally validated in multiple large registries, uses five variables—age, creatinine clearance, haemoglobin, white blood cell count, and prior bleeding—to estimate bleeding risk during DAPT [46]. A score of 25 or above identifies patients in whom extended DAPT is more likely to cause harm than benefit, supporting an abbreviated therapy of three to six months.

The DAPT score was developed to predict the benefit of extending DAPT beyond 12 months [47]. It incorporates age, smoking status, diabetes, MI at presentation, prior PCI or MI, use of paclitaxel-eluting stents, stent diameter, and other variables. A score of 2 or above identifies patients likely to benefit from prolonged therapy, whilst a score below 2 favours discontinuation.

The ARC-HBR criteria provide a standardised consensus-based definition of high bleeding risk, developed specifically for use in clinical trials and practice [13]. The framework categorises bleeding risk factors into major (anticipated long-term oral anticoagulation, severe or end-stage chronic kidney disease [CKD], liver cirrhosis with portal hypertension, haemoglobin < 11 g/dL, among others) and minor (age ≥ 75 years, moderate CKD, chronic NSAID or corticosteroid use) criteria. One major criterion or two minor criteria define HBR status [13]. The ARC-HBR criteria underpin the patient selection in MASTER-DAPT and have been adopted as a practical decision-support tool in both ESC and ACC/AHA guidelines. The key features, variables, and clinical applications of validated risk stratification tools are compared in Table 2.

The PRECISE-HBR score, developed in 2024–2025 by integrating the PRECISE-DAPT score with the ARC-HBR criteria, represents a more contemporary approach to bleeding risk assessment specifically calibrated to current PCI practice [48]. PRECISE-HBR combines the laboratory and demographic variables of PRECISE-DAPT with the major and minor criteria of ARC-HBR, yielding a continuous score that has undergone both internal validation in the derivation cohort and external validation in independent registries. It outperforms either parent instrument used alone for the prediction of out-of-hospital BARC 3 or 5 bleeding within 12 months of PCI and offers a more granular stratification across the bleeding-risk spectrum, which is particularly relevant for tailoring DAPT duration in patients who do not fit neatly into the binary ARC-HBR classification. Its incorporation alongside PRECISE-DAPT, the DAPT score and ARC-HBR criteria is now reasonable in routine practice.

Dynamic reassessment is essential. Bleeding risk is not static: intercurrent anaemia, worsening renal function, initiation of oral anticoagulation, or any bleeding episode should trigger reassessment of the antiplatelet plan. Emerging tools—including machine learning models incorporating high-sensitivity troponin, D-dimer, and platelet-derived biomarkers—show promise for refining risk prediction but remain exploratory [49].

**Figure 2:**
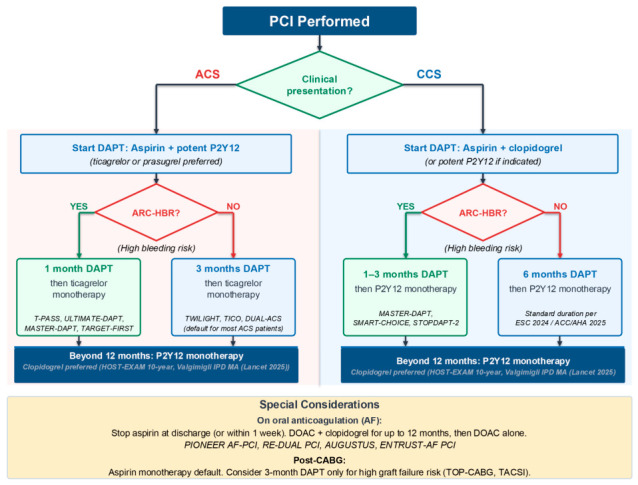
A Practical Framework for Individualising Post-PCI Antiplatelet Therapy.

An integrated clinical decision framework for antiplatelet selection after PCI, incorporating clinical presentation and ARC-HBR status, is provided in Figure 2.

## 9. Special Clinical Contexts

### 9.1. Concomitant Oral Anticoagulation (Atrial Fibrillation and PCI)

In patients requiring long-term oral anticoagulation, such as those with atrial fibrillation (AF), the combination of DAPT with an oral anticoagulant (triple therapy) substantially increases bleeding risk without a proportional reduction in thromboembolic events. Four landmark trials—PIONEER AF-PCI, RE-DUAL PCI, AUGUSTUS, and ENTRUST-AF PCI—have established that dual therapy with a direct oral anticoagulant (DOAC) plus a P2Y_12_ inhibitor, with aspirin stopped at or shortly after PCI, significantly reduces bleeding compared with warfarin-based triple therapy whilst maintaining ischaemic safety [50,51,52,53].

PIONEER AF-PCI demonstrated that rivaroxaban plus clopidogrel significantly reduced clinically significant bleeding compared with warfarin-based triple therapy (HR 0.59, 95% CI 0.47–0.76), with similar rates of major adverse cardiovascular events across groups [50]. RE-DUAL PCI showed that dabigatran-based dual therapy reduced major or clinically relevant non-major bleeding compared with triple therapy (HR 0.52, 95% CI 0.42–0.63 for 110 mg; HR 0.72, 95% CI 0.58–0.88 for 150 mg), with a non-inferior efficacy composite [51]. AUGUSTUS, a 2 × 2 factorial trial of apixaban versus vitamin K antagonist and aspirin versus placebo, confirmed that aspirin-free regimens significantly reduced bleeding (HR 0.69, 95% CI 0.58–0.81) without increasing ischaemic events [52]. ENTRUST-AF PCI provided concordant data with edoxaban-based dual therapy [53].

Current guidance recommends stopping aspirin at hospital discharge or within one week of PCI in patients requiring oral anticoagulation, with DOAC plus clopidogrel as the preferred dual therapy for up to 12 months, followed by DOAC monotherapy [6,45]. Potent P2Y_12_ inhibitors (ticagrelor, prasugrel) are avoided in combination with OACs owing to an increased risk of bleeding.

### 9.2. Coronary Artery Bypass Grafting

Aspirin monotherapy remains the standard antiplatelet strategy for most patients following CABG, supported by longstanding guideline recommendations [54]. The optimal duration of DAPT after CABG, however, has been less clearly defined, with mechanistic and observational data suggesting that the risk of early saphenous vein graft stenosis is highest within the first three months after surgery.

Results from TOP-CABG suggest that a de-escalation strategy of three months of DAPT (ticagrelor plus aspirin) followed by aspirin monotherapy may be viable [55]. Graft occlusion appeared non-inferior (10.79% vs. 11.19%), and bleeding was substantially lower (8.26% vs. 13.19%; HR 0.62) [55]. TACSI, a published randomised trial in 2201 ACS patients requiring CABG, compared ticagrelor plus aspirin with aspirin alone and found similar MACE rates (4.8% vs. 4.6%; HR 1.06, 95% CI 0.72–1.56, *p* = 0.77) but a twofold increase in major bleeding with DAPT (4.9% vs. 2.0%; HR 2.50) [56]. These findings highlight an important tension: TOP-CABG suggests a short DAPT course may protect vein grafts without unacceptable bleeding in selected patients, whilst TACSI demonstrates that DAPT confers no MACE advantage over aspirin monotherapy in ACS patients undergoing CABG and substantially increases bleeding. Taken together, these data support aspirin monotherapy as the default antiplatelet strategy after CABG. A limited course of DAPT (approximately three months) may be considered in patients perceived to be at particularly high vein graft failure risk—for example, those with multiple sequential vein grafts, diffuse coronary disease, or incomplete revascularisation—although it must be acknowledged that no validated clinical tool currently exists to identify such patients prospectively. The clinical decision to use post-CABG DAPT should therefore be individualised, weighed explicitly against bleeding risk, and revisited in light of further trial evidence. TOP-CABG full results remain in conference-abstract form pending peer-reviewed publication, whereas the full TACSI dataset has been published [56].

A related and clinically important caveat applies to long-term P2Y_12_ monotherapy. When a patient on antiplatelet monotherapy requires non-cardiac surgery, whether elective or urgent, the perioperative bleeding profile of aspirin is comparatively well characterised: continuation of low-dose aspirin in most non-cardiac surgical settings does not confer a clinically meaningful increase in perioperative bleeding above the small absolute baseline, and withdrawal carries a measurable cardiovascular cost [57]. The corresponding evidence base for clopidogrel, and a fortiori for ticagrelor, is considerably less mature, and many surgical and anaesthetic teams remain reluctant to operate on patients receiving these agents without an interruption or a bridge to aspirin. As a consequence, patients on long-term clopidogrel monotherapy may, in practice, be transitioned back to aspirin in advance of an elective surgical procedure, and this transition itself carries pharmacodynamic risk if mistimed. Clinicians selecting between aspirin and clopidogrel monotherapy for chronic secondary prevention should therefore factor in the patient’s anticipated need for subsequent surgery and the comfort of the local surgical and anaesthetic teams with P2Y_12_-inhibitor management, in addition to the underlying ischaemic and bleeding risk profile.

### 9.3. High Bleeding Risk Patients

Elderly patients (aged over 75 years) are disproportionately affected by major bleeding on prolonged DAPT, and the MASTER-DAPT trial provides the most relevant evidence for this population [33]. Abbreviated DAPT (one to three months) is consistently supported in HBR patients across MASTER-DAPT, TWILIGHT HBR subgroup analyses, and serial data from the STOPDAPT programme. In STOPDAPT-3, the HBR subgroup with ACS showed that even in patients selected for high bleeding risk, aspirin withdrawal at PCI failed to reduce major bleeding and was associated with excess MI, reinforcing the principle that timing of aspirin cessation matters independently of bleeding risk category [22]. Beyond age, frailty, polypharmacy (particularly concurrent use of non-steroidal anti-inflammatory drugs or corticosteroids), and concomitant oral anticoagulation—frequently co-existing in this population—each independently increase bleeding risk and should trigger consideration of abbreviated DAPT. The ARC-HBR criteria serve as a practical clinical tool for identifying these patients at the point of PCI, and their adoption in both ESC and ACC/AHA guidelines reflects the growing consensus that HBR status should guide DAPT duration rather than a fixed default [6,13,45].

### 9.4. Chronic Kidney Disease and Diabetes

Both CKD and diabetes mellitus increase thrombotic risk after PCI, and both present complex interactions with antiplatelet therapy [58,59]. In CKD, uraemia produces profound alterations in haemostasis characterised by platelet dysfunction alongside paradoxical platelet activation, resulting in a simultaneous predisposition to both bleeding and thrombosis. The net consequence for antiplatelet therapy is that these patients face elevated risk from both excessive antithrombotic intensity and insufficient platelet inhibition—a clinical paradox that standard risk scores do not fully capture. Patients with diabetes demonstrate persistent platelet hyperreactivity and may exhibit attenuated response to clopidogrel through mechanisms that include, but are not limited to, CYP2C19 variability [60]. This latter interaction is of direct relevance when aspirin is withdrawn and antithrombotic protection relies entirely on P2Y_12_ inhibition: diabetic patients who carry CYP2C19 loss-of-function alleles and are maintained on clopidogrel monotherapy may be left with inadequate platelet inhibition, particularly in the higher-risk period immediately after PCI for ACS.

Subgroup analysis from the DAPT Trial suggested a potential reduction in ischaemic events with DAPT beyond 12 months among diabetic patients (HR 0.72, 95% CI 0.54–0.97) [4]. Subsequent meta-analyses, however, have not consistently demonstrated improved cardiovascular outcomes with prolonged DAPT in patients with diabetes or CKD [61]. These observations reflect the fundamental challenge of these populations: the evidence base is largely derived from subgroup analyses and observational cohorts rather than adequately powered, prospectively designed trials. The available evidence does not support routine prolongation of aspirin-containing DAPT solely on the basis of diabetes or CKD. What it does support is heightened vigilance: in both populations, the choice of P2Y_12_ agent, the assessment of CYP2C19 genotype where relevant, and the dynamic reassessment of bleeding and ischaemic risk over time are more important than a fixed DAPT duration. Dedicated trials in patients with advanced CKD or poorly controlled diabetes—populations systematically excluded or underrepresented in most de-escalation trials—represent an important unmet need.

## 10. Beyond One Year: Long-Term Monotherapy

Once the first 12 months of post-PCI therapy are navigated uneventfully, the clinical question shifts from ‘how long is DAPT used?’ to ‘which agent is used as monotherapy?’ Emerging evidence increasingly favours P2Y_12_ inhibitors over aspirin for chronic secondary prevention.

The PANTHER meta-analysis pooled data from seven randomised trials (*n* = 24,325; 60.6% ACS) comparing P2Y_12_ inhibitor monotherapy with aspirin monotherapy over approximately two years [62]. The primary composite of cardiovascular death, MI, and stroke was significantly reduced with P2Y_12_ inhibitors (5.5% vs. 6.3%; HR 0.88, 95% CI 0.79–0.97), largely driven by fewer MIs. Major bleeding was similar between groups (1.2% vs. 1.4%), and NACE favoured P2Y_12_ monotherapy (6.4% vs. 7.2%) [62].

HOST-EXAM Extended provides the longest-duration randomised comparison of clopidogrel versus aspirin monotherapy after PCI [63]. In the extended follow-up of the original HOST-EXAM cohort (5438 patients who remained event-free for 12 ± 6 months after DES implantation), clopidogrel monotherapy was associated with significantly better net clinical outcomes over a median follow-up of 5.8 years (HR 0.74, 95% CI 0.63–0.86), with lower thrombotic endpoints (HR 0.66, 95% CI 0.55–0.79) and lower major bleeding (BARC ≥ 2: HR 0.74, 95% CI 0.57–0.94) [63]. Landmark analysis at two years confirmed that the clinical benefit of clopidogrel persisted throughout extended follow-up. This is the strongest long-term evidence favouring clopidogrel over aspirin as chronic secondary prevention after PCI.

A 2025 individual patient data (IPD) meta-analysis by Giacoppo and colleagues pooled five randomised trials (*n* = 16,117; median follow-up 5.5 years) comparing P2Y_12_ monotherapy with aspirin monotherapy after DAPT completion [64]. MACCE was significantly reduced with P2Y_12_ monotherapy (HR 0.77, 95% CI 0.67–0.89; number needed to treat approximately 45), with significant reductions in both MI and stroke. Major bleeding HR ranged from 1.12 to 1.26, but all confidence intervals crossed unity (*p* > 0.3), indicating no significant bleeding difference [64]. These IPD-level data provide the most comprehensive evidence to date that P2Y_12_ monotherapy is the superior long-term antiplatelet strategy after PCI.

Two further individual patient data (IPD) meta-analyses have refined the evidence base in this area and warrant explicit consideration. An IPD meta-analysis of de-escalation to ticagrelor monotherapy versus 12 months of dual antiplatelet therapy in patients with and without ACS, published in The Lancet in 2024, pooled patient-level data from the major ticagrelor-based aspirin-withdrawal trials [38]. Ticagrelor monotherapy after a brief initial DAPT period reduced major bleeding without excess in the composite of cardiovascular death, MI or stroke, including in pre-specified ACS and complex PCI subgroups. Because the analysis was specific to ticagrelor, it does not directly support the same conclusion for clopidogrel monotherapy in this early-phase setting. A separate IPD meta-analysis published in The Lancet in 2025 then compared clopidogrel monotherapy with aspirin monotherapy for long-term secondary prevention of coronary artery disease [65]. Pooled across multi-year follow-up, clopidogrel monotherapy reduced the composite of cardiovascular death, MI and stroke without significant excess in major bleeding. Taken together with PANTHER, HOST-EXAM Extended and the BMJ 2025 IPD meta-analysis, these data converge on a coherent picture: in event-free patients who have completed an initial period of DAPT, ticagrelor monotherapy is the more pharmacogenomically robust early-phase option, and clopidogrel monotherapy is at least as safe as aspirin monotherapy in the chronic phase, with a more favourable bleeding profile.

The 10-year extended follow-up of the HOST-EXAM trial, published in The Lancet in 2026, now provides the longest-duration randomised comparison of clopidogrel versus aspirin monotherapy after PCI [66]. In 5438 patients who had completed an initial period of DAPT after PCI and were event-free at randomisation, the cohort was followed for a median of 10.5 years with a 92.8% completion rate. The primary composite endpoint of all-cause death, non-fatal myocardial infarction, stroke, readmission for acute coronary syndrome and BARC type 3 or higher bleeding occurred in 25.4% of the clopidogrel arm versus 28.5% of the aspirin arm (HR 0.86, 95% CI 0.77–0.96; *p* = 0.0050). The thrombotic component was lower with clopidogrel (17.3% vs. 20.0%; *p* = 0.0024), as was the bleeding component (9.1% vs. 10.8%; *p* = 0.020), and all-cause mortality was similar between arms. These 10-year findings reaffirm and extend the original 2-year HOST-EXAM and 5-year HOST-EXAM Extended results [63] to a follow-up horizon that no other trial in this field approaches. Together with the STOPDAPT-2 5-year landmark analysis [37], the consistency of the cardiovascular benefit of clopidogrel over aspirin from one year out to ten years, without excess major bleeding, is now the strongest single body of evidence supporting P2Y_12_ monotherapy as default chronic secondary prevention after PCI in event-free patients.

A 23-trial network meta-analysis (*n* = 45,394) further contextualised these findings by comparing multiple post-PCI antiplatelet strategies [67]. P2Y_12_ monotherapy versus aspirin monotherapy yielded a NACE incidence rate ratio of 0.78 (95% CI 0.64–0.95) and any-bleeding incidence rate ratio of 0.68 [67]. Aspirin monotherapy after short DAPT was neutral for NACE compared with standard DAPT. P2Y_12_ monotherapy was the only strategy that reduced both NACE and bleeding—a finding of direct clinical relevance.

For selected patients at very high persistent ischaemic risk and low bleeding risk, PEGASUS–TIMI 54 demonstrated that prolonged DAPT with ticagrelor (60 mg or 90 mg) plus aspirin significantly reduced the composite of cardiovascular death, MI, or stroke compared with placebo over approximately 33 months (HR 0.84–0.85) [68]. This ischaemic benefit, however, was accompanied by a significant increase in TIMI major bleeding (2.30–2.60% vs. 1.06%), indicating that extended DAPT should be reserved for carefully selected patients in whom the residual ischaemic risk clearly justifies the additional bleeding burden.

Taken together, these data support a fundamental shift in long-term secondary prevention after PCI. For many patients who remain event-free after the first year, P2Y_12_ monotherapy—preferably clopidogrel, given its tolerability and cost profile—appears to be a reasonable and potentially superior long-term antiplatelet strategy. The routine continuation of aspirin as lifelong therapy is increasingly difficult to justify in light of this evidence.

## 11. Methodological Considerations

Interpretation of contemporary evidence on abbreviated DAPT and early aspirin withdrawal after PCI must account for several methodological features common to many pivotal trials in this field.

A substantial proportion of de-escalation trials have been structured as non-inferiority studies, and this design choice has direct interpretive consequences. Non-inferiority is appropriate when the anticipated benefit relates to reduced bleeding rather than improved efficacy, and the objective is to exclude a clinically unacceptable increase in adverse outcomes. Trials including SMART-CHOICE, STOPDAPT-2, TICO and MASTER-DAPT all adopted this framework. By contrast, only a minority of trials in this field have been powered for and have demonstrated formal superiority—for example STOPDAPT-2 for net adverse clinical events in a mixed population, T-PASS for NACE in ACS, and HOST-EXAM Extended for long-term clopidogrel over aspirin monotherapy. The distinction matters for guideline translation: non-inferiority on a composite of bleeding plus ischaemia does not establish that an abbreviated strategy is therapeutically equivalent across endpoints, only that the prespecified margin was met. Interpretation depends heavily on the selected non-inferiority margin; permissive margins may admit modest increases in thrombotic events while still meeting statistical criteria. Absolute event rates, the direction and magnitude of point estimates, and component-level effects should always be examined alongside hazard ratios to assess clinical meaningfulness.

The incorporation of run-in phases prior to randomisation is another important design feature with implications for external validity. TWILIGHT provides the clearest example: patients who experienced major bleeding or ischaemic events during the initial three-month DAPT period were excluded from randomisation [30]. This approach strengthens internal validity by ensuring that the randomised population is clinically stable and treatment-tolerant. It also, however, systematically excludes the patients at greatest early risk—precisely those in whom the bleeding–ischaemia trade-off is most consequential. Because the run-in selects an enriched, lower-risk cohort, the subsequent comparison may overestimate the safety of aspirin withdrawal and underestimate the absolute event rates a clinician will encounter in unselected real-world practice. The same caveat applies, to varying degrees, to ULTIMATE-DAPT, TICO, T-PASS and TARGET-FIRST, all of which randomised only event-free patients after an initial DAPT period. The aggregate trial estimate of bleeding reduction is therefore best read as the upper bound of benefit achievable in a stable, fully compliant patient who has tolerated initial DAPT, not the benefit expected if the same regimen were applied indiscriminately at hospital discharge.

The demographic composition of trial populations warrants careful consideration. Several pivotal trials, including STOPDAPT-2 (exclusively Japanese), SMART-CHOICE (predominantly Korean), and HOST-EXAM Extended (exclusively Korean), were conducted in East Asian populations [34,35,63]. East Asian patients tend to experience lower rates of ischaemic complications following PCI but demonstrate increased vulnerability to bleeding when exposed to antithrombotic therapy. Variations in platelet reactivity, pharmacogenomic influences affecting drug metabolism, and differences in baseline cardiovascular risk profiles may all contribute to these observations. The favourable safety profile associated with abbreviated DAPT in these cohorts may not be directly generalisable to populations with a higher burden of atherothrombotic risk. In practical terms, this means that absolute bleeding reductions reported from STOPDAPT-2, SMART-CHOICE, TICO, T-PASS and HOST-EXAM Extended cannot be transposed directly to European or North American practice without adjustment for the higher underlying ischaemic burden and lower baseline bleeding rates of those populations. Where transethnic confirmation has been sought, for example in TWILIGHT, MASTER-DAPT, ULTIMATE-DAPT and the European arms of DUAL-ACS, the qualitative direction of effect has generally been preserved, but the absolute risk differences are typically smaller than in East Asian-only cohorts.

Composite endpoints combining ischaemia and bleeding in a single net adverse clinical event (NACE) metric can obscure clinically important component-level trade-offs. Several pivotal trials in this field have generated headline NACE results that are driven almost entirely by reductions in nuisance bleeding (BARC 2) rather than by balanced effects across all components, with TWILIGHT, TICO and MASTER-DAPT as illustrative examples. A strategy that halves bleeding but doubles the risk of a rare but consequential ischaemic event (stent thrombosis, spontaneous MI) may appear neutral or favourable on a composite while having materially different implications at the individual patient level. Equally, in NEO-MINDSET and STOPDAPT-3, the directional increase in stent thrombosis and spontaneous MI was numerically modest but clinically weighted, and component-level inspection is essential to surface such signals. Reporting that explicitly disaggregates the composite, weights events by clinical severity, and provides absolute event rates is therefore preferable to reliance on the composite hazard ratio alone.

Concomitant proton pump inhibitor (PPI) use varies across trials and directly affects gastrointestinal bleeding event rates, yet is rarely fully accounted for in primary analyses. Differences in PPI co-prescription between arms or across trial populations may confound bleeding comparisons.

Finally, many DAPT de-escalation trials, including DUAL-ACS and TWILIGHT, adopted open-label designs, which are susceptible to performance and detection bias, particularly for softer ischaemic endpoints such as unplanned revascularisation [30,32]. Blinded trials such as ULTIMATE-DAPT provide the most rigorous test of aspirin withdrawal strategies [26].

A further consideration that is rarely addressed explicitly in this literature is publication bias and selective reporting. The abbreviated DAPT trial landscape is characterised by a predominance of neutral or positive results, and several trials have received partial or full funding from device or pharmaceutical manufacturers with a commercial interest in shorter DAPT durations. Although this does not invalidate individual findings, it warrants acknowledgement. Funnel plot asymmetry has been noted in some network meta-analyses of DAPT duration trials, suggesting that smaller, negative studies may be underrepresented in the published literature. Clinicians interpreting the aggregate evidence should bear this in mind, particularly when considering the generalisability of effect sizes from selected trial populations to unselected clinical practice.

## 12. Future Directions

Several developments may further refine post-PCI antiplatelet strategies in the coming years.

Selatogrel, a subcutaneous P2Y_12_ antagonist with a rapid onset of action, is under phase 3 evaluation in the SOS-AMI trial (NCT04957719), which is assessing self-administration at symptom onset in patients at risk of recurrent AMI. Its pharmacokinetic profile—bypassing the need for oral absorption and hepatic activation—may make it particularly suited to pre-hospital ACS management or self-administered rescue therapy in patients who cannot take oral antiplatelet agents [69].

BMS-986141, a protease-activated receptor 4 (PAR-4) inhibitor, has completed early-phase evaluation in stable coronary artery disease, demonstrating dose-dependent inhibition of PAR-4-triggered platelet aggregation. Combining PAR-4 antagonism with very-low-dose factor Xa inhibition shows additive antithrombotic effects without disproportionate bleeding in preclinical models, raising the possibility of entirely novel antithrombotic combinations that do not depend on aspirin or conventional P2Y_12_ inhibition [70].

Ultra-short DAPT meta-analyses published in 2025, pooling trials of one month or less of DAPT versus six months or more, demonstrated approximately 20% reductions in NACE and approximately 50% reductions in clinically relevant bleeding [71]. These analyses, however, are dominated by East Asian populations, and the routine application of ultra-short DAPT in ethnically diverse cohorts remains premature pending further data.

Imaging-guided PCI—with intravascular ultrasound (IVUS) or optical coherence tomography (OCT)—has shown improved stent deployment optimisation in OCTOBER and ILUMIEN IV [72,73]. By ensuring adequate stent expansion, apposition, and lesion coverage, imaging-guided PCI may further reduce early thrombotic risk, potentially narrowing the window during which dual antiplatelet therapy is required.

Precision medicine approaches combining pharmacogenomic profiling (CYP2C19), platelet function testing, and machine learning risk models may enable truly individualised antiplatelet selection. None of these tools is yet ready for routine clinical integration, but the trajectory is clear: the future of post-PCI antiplatelet therapy is unlikely to be a single, uniformly applied protocol, but rather a framework that adapts agents, doses, and durations to the individual patient.

## 13. Conclusions

The evidence reviewed here supports a fundamental re-evaluation of aspirin’s role after PCI, structured around timing and clinical context rather than a uniform prescription.

In the immediate post-PCI period (first two to four weeks), aspirin remains indispensable. NEO-MINDSET and STOPDAPT-3 demonstrated an early signal of excess ischaemic events when aspirin was withdrawn at PCI in ACS populations, confirming that aspirin remains important during the earliest post-procedural period.

After one month, in patients who are event-free, transition to ticagrelor (or prasugrel) monotherapy reduces bleeding without measurable ischaemic cost in selected populations (T-PASS, ULTIMATE-DAPT, TARGET-FIRST). Three-month DAPT, supported by the largest body of randomised evidence (TWILIGHT, TICO, DUAL-ACS), appears to offer the most favourable trade-off between efficacy and safety for a broad ACS population.

Beyond 12 months, P2Y_12_ monotherapy outperforms aspirin as chronic secondary prevention. For patients with stable coronary disease or those who are genotype-confirmed normal CYP2C19 metabolisers, clopidogrel is a reasonable first choice given its tolerability and cost profile. In patients with prior ACS who are not genotype-tested, ticagrelor or prasugrel monotherapy should be considered given clopidogrel’s pharmacogenomic variability. The 2025 BMJ individual patient data meta-analysis [64] and HOST-EXAM Extended [63] provide the strongest long-term data supporting this shift.

Risk stratification using PRECISE-DAPT, the DAPT score, ARC-HBR criteria and the more recent PRECISE-HBR score [48], combined with dynamic reassessment of bleeding and ischaemic risk, is essential for individualising both DAPT duration and monotherapy selection. The choice of P2Y_12_ agent remains critical: ticagrelor and prasugrel are preferred for aspirin-free strategies in ACS, whereas clopidogrel may be appropriate in genotype-confirmed normal metabolisers, stable coronary disease settings, or patients with contraindications to potent P2Y_12_ inhibitors. The 10-year HOST-EXAM follow-up [66] and the STOPDAPT-2 5-year landmark analysis [37] now provide consistent randomised evidence that clopidogrel monotherapy outperforms aspirin monotherapy for chronic secondary prevention beyond the first post-PCI year, without excess major bleeding. The era of uniform, lifelong aspirin after PCI is therefore giving way to a personalised strategy in which aspirin retains an essential role in the early post-procedural window and in defined long-term subgroups, but in which time-limited and patient-specific de-escalation to P2Y_12_ monotherapy is increasingly the default for event-free patients who have completed an initial period of DAPT.

## Figures and Tables

**Table 1:** Summary of randomised trials evaluating aspirin withdrawal and antiplatelet de-escalation strategies after PCI. Trials are organised by timing of aspirin cessation: immediate withdrawal (0 months), short-duration DAPT (1 month), intermediate-duration DAPT (3 months), extended DAPT beyond one year, and special populations (oral anticoagulation, CABG). ACS, acute coronary syndrome; CABG, coronary artery bypass grafting; CCS, chronic coronary syndrome; CI, confidence interval; DAPT, dual antiplatelet therapy; HR, hazard ratio; NACE, net adverse clinical events; PCI, percutaneous coronary intervention; RR, relative risk.

Trial	Year	Design	Population	N	Aspirin Strategy	P2Y_12_ Agent	Comparator	Primary Endpoint	Key Findings
***Immediate Aspirin Withdrawal (at PCI)***									
STOPDAPT-3	2024	RCT	ACS + HBR	5966	Immediate stop	Prasugrel	DAPT	CV composite + bleeding	Non-inferior for CV composite; failed to reduce bleeding. Excess stent thrombosis (HR 3.40) and revascularisation
NEO-MINDSET	2025	RCT	ACS	3410	Stop within 4 days	Potent P2Y_12_	DAPT	Ischaemic composite (12 mo)	Failed non-inferiority for ischaemia (7.0% vs. 5.5%). Bleeding ↓ (2.0% vs. 4.9%). STEMI substudy: HR 1.60 for ischaemia
***Aspirin Withdrawal at One Month***									
T-PASS	2023	RCT	ACS	2850	Stop ~16 days	Ticagrelor	12-mo DAPT	NACE	NACE ↓ (2.8% vs. 5.2%; HR 0.54). Major bleeding halved, no excess ischaemia. East Asian only.
ULTIMATE-DAPT	2024	RCT	ACS (high-risk)	~3400	Stop at 1 month	Ticagrelor	Ticagrelor + ASA	Bleeding (mo 1–12)	Clinically relevant bleeding ↓ (2.1% vs. 4.6%; HR 0.45). MACCE non-inferior (3.6% vs. 3.7%). Double-blind.
TARGET-FIRST	2025	RCT	Low-risk AMI, complete revasc	1942	Stop at 1 month	P2Y_12_ mono	DAPT	Ischaemic + bleeding	Ischaemic non-inferiority met. BARC 2/3/5 bleeding halved (2.6% vs. 5.6%; HR 0.46; *p* = 0.002).
GLOBAL LEADERS	2018	RCT	All-comers PCI	15,968	Stop at 1 month	Ticagrelor	Standard DAPT → ASA	Death or Q-wave MI (24 mo)	Neutral (RR 0.87, CI 0.75–1.01). No early bleeding benefit. Heterogeneous population may have diluted effect.
***Three-Month DAPT Strategies***									
TWILIGHT	2019	RCT	High-risk PCI	7119	Stop at 3 months	Ticagrelor	Ticagrelor + ASA	BARC 2–5 bleeding	BARC 2–5 ↓ 44% (4.0% vs. 7.1%; HR 0.56; NNT ~32). Death/MI/stroke non-inferior (3.9% vs. 3.9%).
TICO	2020	RCT	ACS	~3000	Stop at 3 months	Ticagrelor	12-mo DAPT	NACE	NACE ↓, driven by lower bleeding. Ischaemic outcomes non-inferior. East Asian only.
DUAL-ACS	2025	RCT	AMI	5052	Stop at 3 months	P2Y_12_ mono	12-mo DAPT	All-cause death + bleeding	Trend ↓ mortality (HR 0.78; *p* = 0.12) and ↓ major bleeding (HR 0.78; *p* = 0.10). Largest pragmatic MI trial.
MASTER-DAPT	2021	RCT	ARC-HBR	4579	Stop at 1–3 months	P2Y_12_ (mainly clopi)	Prolonged DAPT	NACE	Non-inferior ischaemia. Clinically relevant bleeding ↓ (absolute diff −2.82 pp; *p* < 0.001).
SMART-CHOICE	2019	RCT	Mainly CCS	~3000	Stop at 3 months	Mainly clopidogrel	12-mo DAPT	MACCE	Non-inferior ischaemic outcomes. East Asian only.
STOPDAPT-2	2019	RCT	CCS + ACS	3045	Stop at 1 month	Clopidogrel	12-mo DAPT	NACE	Superior NACE (2.4% vs. 3.7%; HR 0.64), driven by bleeding ↓. ACS subgroup later showed excess MI (see below).
***The Clopidogrel Problem***									
STOPDAPT-2 ACS	2022	RCT	ACS	4136	Stop at 1–2 months	Clopidogrel	12-mo DAPT	NACE	Failed non-inferiority (HR 1.14; *p* = 0.06 for NI). ↑ CV events (2.8% vs. 1.9%). Clopidogrel monotherapy unsafe in ACS.
POPular Genetics	2019	RCT	STEMI	2488	Genotype-guided selection	Guided (clopi/tica/pras)	Standard (pras/tica)	Thrombotic + bleeding composite	Non-inferior (5.1% vs. 5.9%). Bleeding ↓ (9.8% vs. 12.5%; HR 0.78). Supports genotype-guided selection.
***Tailored De-escalation Strategies***									
4D-ACS	2025	RCT	ACS	1370	1-mo DAPT → prasugrel 5 mg mono	Prasugrel 5 mg	12-mo DAPT	NACE	NACE ↓ (4.9% vs. 8.8%). BARC 2–5 ↓ 77%. Combines aspirin withdrawal + dose de-escalation.
HOST-REDUCE	2020	RCT	ACS	2338	Prasugrel 10 → 5 mg de-escalation	Prasugrel 5 mg	Prasugrel 10 mg	NACE	BARC 2–5 ↓ (HR 0.48). Ischaemic non-inferiority met. Supports P2Y_12_ dose reduction.
OPT-BIRISK	2024	RCT	ACS (dual high risk)	7758	Extended clopi mono (9 mo post-DAPT)	Clopidogrel	DAPT	BARC 2/3/5 + MACCE	Bleeding ↓ (2.5% vs. 3.3%; HR 0.75). MACCE ↓ (2.6% vs. 3.5%; HR 0.74). Benefits dual-risk patients.
TROPICAL-ACS	2017	RCT	ACS	2610	Guided de-escalation (pras → clopi)	Guided clopidogrel	Prasugrel	Ischaemic + bleeding composite	Non-inferior. Proof-of-concept for platelet function-guided de-escalation.
***Beyond One Year: Long-Term Monotherapy***									
HOST-EXAM Extended	2023	RCT	Post-DES (event-free)	5438	Clopidogrel vs. aspirin mono	Clopidogrel	Aspirin	Net clinical outcome (5.8 yr)	Clopidogrel superior (HR 0.74). ↓ Thrombotic events (HR 0.66) and ↓ major bleeding (HR 0.74). Longest RCT comparison.
HOST-EXAM 10-yr	2026	RCT (10-yr extended follow-up)	Post-DES, event-free at randomisation	5438	Aspirin mono	Clopidogrel	Aspirin	Composite (death/MI/stroke/ACS readmission/BARC ≥ 3) over 10.5 yr	92.8% completion. Primary 25.4% vs. 28.5% (HR 0.86, 95% CI 0.77–0.96; *p* = 0.0050). Thrombotic 17.3% vs. 20.0% (*p* = 0.0024). Bleeding 9.1% vs. 10.8% (*p* = 0.020). All-cause mortality similar. Now the longest-duration RCT comparison of clopidogrel vs. aspirin monotherapy after PCI.
STOPDAPT-2 5-yr	2026	RCT (5-yr landmark; year 1–5 follow-up)	Post-PCI; ACS subgroup + total cohort	5991 (total)/2986 (ACS)	Aspirin mono after year 1	Clopidogrel mono	Aspirin mono	CV composite + bleeding	ACS cohort: primary composite ↓ 6.18% vs. 8.27% (HR 0.75, 95% CI 0.57–0.997; *p* = 0.048); CV composite ↓ (HR 0.70, 95% CI 0.51–0.96; *p* = 0.03). Total cohort: CV ↓ (HR 0.74, 95% CI 0.60–0.91; *p* = 0.004); no difference in TIMI major/minor bleeding. Establishes clopidogrel superiority over aspirin for chronic monotherapy beyond year 1.
PANTHER (meta-analysis)	2023	IPD MA	60.6% ACS	24,325	P2Y_12_ vs. aspirin mono	P2Y_12_ (various)	Aspirin	CV death/MI/stroke	P2Y_12_ superior (5.5% vs. 6.3%; HR 0.88). NACE favoured P2Y_12_ (6.4% vs. 7.2%).
BMJ IPD MA (Giacoppo)	2025	IPD MA	Post-DAPT PCI	16,117	P2Y_12_ vs. aspirin mono (5.5 yr)	P2Y_12_ (various)	Aspirin	MACCE	MACCE ↓ (HR 0.77; NNT ~45). ↓ MI and stroke. No significant bleeding difference.
PEGASUS–TIMI 54	2015	RCT	Prior MI (1–3 yr)	21,162	Extended DAPT (tica 60/90 + ASA)	Ticagrelor	Placebo + ASA	CV death/MI/stroke	CV composite ↓ (HR 0.84–0.85). TIMI major bleeding ↑ (2.3–2.6% vs. 1.1%). For selected very-high-risk patients only.

Abbreviations: ACS, acute coronary syndrome; AMI, acute myocardial infarction; ASA, aspirin; BARC, Bleeding Academic Research Consortium; CCS, chronic coronary syndrome; clopi, clopidogrel; CV, cardiovascular; DAPT, dual antiplatelet therapy; DES, drug-eluting stent; HBR, high bleeding risk; HR, hazard ratio; IPD MA, individual patient data meta-analysis; MACCE, major adverse cardiovascular and cerebrovascular events; MI, myocardial infarction; mo, month(s); NACE, net adverse clinical events; NI, non-inferiority; NNT, number needed to treat; pras, prasugrel; RCT, randomised controlled trial; revasc, revascularisation; STEMI, ST-elevation myocardial infarction; tica, ticagrelor; yr, year(s).

**Table 2:** Comparison of validated risk stratification tools for guiding antiplatelet therapy duration after percutaneous coronary intervention. PRECISE-DAPT and DAPT Score are continuous scoring systems that quantify bleeding and ischaemic risk, respectively, whereas ARC-HBR provides a categorical (binary) classification of high bleeding risk based on major and minor criteria. ARC-HBR, Academic Research Consortium for High Bleeding Risk; DAPT, dual antiplatelet therapy; PRECISE-DAPT, PREdicting bleeding Complications In patients undergoing Stent implantation and subsEquent Dual AntiPlatelet Therapy.

Feature	PRECISE-DAPT [46]	DAPT Score [47]	ARC-HBR [13]	PRECISE-HBR [48]
**Purpose**	Predicts bleeding risk during DAPT	Predicts benefit of extending DAPT beyond 12 months	Identifies patients at high bleeding risk (BARC 3–5 and/or ICH) after PCI	Predicts out-of-hospital BARC 3 or 5 bleeding after PCI; integrates PRECISE-DAPT and ARC-HBR
**Timing of use**	At baseline (time of stent insertion)	At 12 months after index event	At baseline (time of stent insertion)	At baseline (time of stent insertion)
**Type**	Continuous numerical score (0–100)	Continuous numerical score (−2 to +10)	Categorical criteria (major + minor)	Continuous numerical score combining clinical, laboratory and ARC-HBR variables
**Key variables**	Age, creatinine clearance, haemoglobin, WBC count, prior bleeding	Age, smoking, diabetes, MI at presentation, prior PCI/MI, paclitaxel stent, stent diameter < 3 mm, CHF/LVEF < 30%, vein graft stent	Major: long-term OAC, severe/end-stage CKD, liver cirrhosis with portal HTN, haemoglobin < 11 g/dL, active malignancy, prior spontaneous ICH, prior ischaemic stroke, thrombocytopaenia. Minor: age ≥ 75, moderate CKD, Hb 11–12.9 (M)/11–11.9 (F), chronic NSAID/steroid use	Variables of PRECISE-DAPT (age, creatinine clearance, haemoglobin, WBC, prior bleeding) plus ARC-HBR major and minor criteria
**High-risk threshold**	Score ≥ 25: shortened DAPT recommended (3–6 months)	Score ≥ 2: continue DAPT beyond 12 months (benefit > risk)	≥1 major criterion OR ≥ 2 minor criteria define HBR; supports abbreviated DAPT (1–3 months)	Higher score: prefer abbreviated DAPT (1–3 months) and aspirin-free de-escalation; consider PRECISE-HBR thresholds defined in derivation cohort
**Low-risk threshold**	Score < 25: standard or prolonged DAPT	Score < 2: stop DAPT at 12 months (risk > benefit)	Not meeting HBR criteria: standard DAPT duration	Lower score: standard DAPT duration acceptable
**Validation**	Derived from 8 RCTs; externally validated in PLATO trial, European PCI registries, PRODIGY	Derived from DAPT Trial; validated in multiple post-PCI cohorts	Consensus-based (ARC); used as enrolment criterion in MASTER-DAPT, ONYX ONE, endorsed by ESC and ACC/AHA guidelines	Derived 2024–2025; internal validation in derivation cohort and external validation in independent registries
**Guideline endorsement**	ESC 2023 ACS and 2024 CCS guidelines (Class IIb for guiding DAPT duration)	ESC 2023 ACS and 2024 CCS guidelines (Class IIb for extended DAPT decision support); ACC/AHA 2025 ACS guidelines	ESC 2023 ACS guidelines (Class I for identifying HBR)	Not yet endorsed in 2023 ESC or 2025 ACC/AHA guidelines (post-dates them); increasingly used in clinical practice
**Key limitation**	Requires laboratory values; may underperform in very elderly or CKD populations	Applied at 12 months (not at baseline); does not guide early DAPT decisions	Binary classification (HBR vs. not); does not quantify absolute bleeding probability	Evidence base is more recent than parent scores; longer-term outcome calibration awaits further validation

Abbreviations: ARC-HBR, Academic Research Consortium for High Bleeding Risk; BARC, Bleeding Academic Research Consortium; CHF, congestive heart failure; CKD, chronic kidney disease; DAPT, dual antiplatelet therapy; Hb, haemoglobin; HBR, high bleeding risk; HTN, hypertension; ICH, intracranial haemorrhage; LVEF, left ventricular ejection fraction; M, male; F, female; NSAID, non-steroidal anti-inflammatory drug; OAC, oral anticoagulant; PCI, percutaneous coronary intervention; RCT, randomised controlled trial; WBC, white blood cell.

## Data Availability

No new data were created or analysed in this study.

## Abbreviations

The following abbreviations are used in this manuscript:

ACS	Acute Coronary Syndrome
ARC-HBR	Academic Research Consortium for High Bleeding Risk
BARC	Bleeding Academic Research Consortium
CABG	Coronary Artery Bypass Grafting
CCS	Chronic Coronary Syndrome
CKD	Chronic Kidney Disease
COX-1	Cyclooxygenase-1
CYP2C19	Cytochrome P450 2C19
DAPT	Dual Antiplatelet Therapy
DES	Drug-Eluting Stent
DOAC	Direct Oral Anticoagulant
HBR	High Bleeding Risk
HR	Hazard Ratio
IPD	Individual Patient Data
LOF	Loss-Of-Function
MACE	Major Adverse Cardiovascular Events
MACCE	Major Adverse Cardiovascular And Cerebrovascular events
MI	Myocardial Infarction
NACE	Net Adverse Clinical Events
NNT	Number Needed To Treat
OAC	Oral Anticoagulant
PCI	Percutaneous Coronary Intervention
STEMI	ST-elevation Myocardial Infarction
TXA_2_	Thromboxane A_2_

## References

[1] Schömig A., Neumann F.J., Kastrati A., Schühlen H., Blasini R., Hadamitzky M., Walter H., Zitzmann-Roth E.-M., Richardt G., Alt E. (1996). A randomised comparison of antiplatelet and anticoagulant therapy after the placement of coronary-artery stents. N. Engl. J. Med..

[2] Mehta S.R., Yusuf S., Peters R.J.G., Bertrand M.E., Lewis B.S., Natarajan M.K., Malmberg K., Rupprecht H.-J., Zhao F., Chrolavicius S. (2001). Effects of pretreatment with clopidogrel and aspirin followed by long-term therapy in patients undergoing percutaneous coronary intervention: The PCI-CURE study. Lancet.

[3] Steinhubl S.R., Berger P.B., Mann J.T., Fry E.T., DeLago A., Wilmer C., Topol E.J., Credo Investigators (2002). Early and sustained dual oral antiplatelet therapy following percutaneous coronary intervention: A randomised controlled trial. JAMA.

[4] Mauri L., Kereiakes D.J., Yeh R.W., Driscoll-Shempp P., Cutlip D.E., Steg P.G., Normand S.-L.T., Braunwald E., Wiviott S.D., Cohen D.J. (2014). Twelve or 30 months of dual antiplatelet therapy after drug-eluting stents. N. Engl. J. Med..

[5] Bertrand M.E., Simoons M.L., Fox K.A., Wallentin L.C., Hamm C.W., McFadden E., De Feyter P.J., Specchia G., Ruzyllo W. (2002). Management of acute coronary syndromes in patients presenting without persistent ST-segment elevation. Eur. Heart J..

[6] Byrne R.A., Rossello X., Coughlan J.J., Barbato E., Berry C., Chieffo A., Claeys M.J., Dan G.-A., Dweck M.R., Galbraith M. (2023). 2023 ESC guidelines for the management of acute coronary syndromes. Eur. Heart J..

[7] Valgimigli M., Bueno H., Byrne R., Collet J.-P., Costa F., Jeppsson A., Jüni P., Kastrati A., Kolh P., Mauri L. (2018). 2017 ESC focused update on dual antiplatelet therapy in coronary artery disease developed in collaboration with EACTS. Eur. Heart J..

[8] Iakovou I., Schmidt T., Bonizzoni E., Ge L., Sangiorgi G.M., Stankovic G., Airoldi F., Chieffo A., Montorfano M., Carlino M. (2005). Incidence, predictors, and outcome of thrombosis after successful implantation of drug-eluting stents. JAMA.

[9] Palmerini T., Benedetto U., Biondi-Zoccai G., Della Riva D., Bacchi-Reggiani L., Smits P.C., Vlachojannis G.J., Jensen L.O., Christiansen E.H., Berencsi K. (2015). Long-term safety of drug-eluting and bare-metal stents: Evidence from a comprehensive network meta-analysis. J. Am. Coll. Cardiol..

[10] Wallentin L., Becker R.C., Budaj A., Cannon C.P., Emanuelsson H., Held C., Horrow J., Husted S., James S., Katus H. (2009). Ticagrelor versus clopidogrel in patients with acute coronary syndromes. N. Engl. J. Med..

[11] Wiviott S.D., Braunwald E., McCabe C.H., Montalescot G., Ruzyllo W., Gottlieb S., Neumann F.-J., Ardissino D., De Servi S., Murphy S.A. (2007). Prasugrel versus clopidogrel in patients with acute coronary syndromes. N. Engl. J. Med..

[12] Eikelboom J.W., Mehta S.R., Anand S.S., Xie C., Fox K.A., Yusuf S. (2006). Adverse impact of bleeding on prognosis in patients with acute coronary syndromes. Circulation.

[13] Urban P., Mehran R., Colleran R., Angiolillo D.J., Byrne R.A., Capodanno D., Cuisset T., Cutlip D., Eerdmans P., Eikelboom J. (2019). Defining high bleeding risk in patients undergoing percutaneous coronary intervention. Circulation.

[14] Patrono C., García Rodríguez L.A., Landolfi R., Baigent C. (2005). Low-dose aspirin for the prevention of atherothrombosis. N. Engl. J. Med..

[15] Dorsam R.T., Kunapuli S.P. (2004). Central role of the P2Y12 receptor in platelet activation. J. Clin. Investig..

[16] Mega J.L., Close S.L., Wiviott S.D., Shen L., Hockett R.D., Brandt J.T., Walker J.R., Antman E.M., Macias W., Braunwald E. (2009). Cytochrome P-450 polymorphisms and response to clopidogrel. N. Engl. J. Med..

[17] Scott S.A., Sangkuhl K., Stein C.M., Hulot J.-S., Mega J.L., Roden D.M., Klein T.E., Sabatine M.S., Johnson J.A., Shuldiner A.R. (2013). Clinical Pharmacogenomics Implementation Consortium guidelines for CYP2C19 genotype and clopidogrel therapy: 2013 update. Clin. Pharmacol. Ther..

[18] Libby P. (2013). Mechanisms of acute coronary syndromes and their implications for therapy. N. Engl. J. Med..

[19] Brambilla M., Camera M., Colnago D., Marenzi G., De Metrio M., Giesen P.L., Balduini A., Veglia F., Gertow K., Biglioli P. (2008). Tissue factor in patients with acute coronary syndromes. Arterioscler. Thromb. Vasc. Biol..

[20] Loeffen R., van Oerle R., Leers M.P.G., Kragten J.A., Crijns H., Spronk H.M.H., Cate H.T. (2016). Factor XIa and thrombin generation are elevated in patients with acute coronary syndrome. PLoS ONE.

[21] Natsuaki M., Watanabe H., Morimoto T., Yamamoto K., Obayashi Y., Nishikawa R., Ando K., Domei T., Suwa S., Ogita M. (2024). An aspirin-free versus dual antiplatelet strategy for coronary stenting: STOPDAPT-3 randomized trial. Circulation.

[22] Ishikawa T., Natsuaki M., Watanabe H., Morimoto T., Yamamoto K., Obayashi Y., Nishikawa R., Ando K., Suwa S., Isawa T. (2025). Aspirin-free strategy for PCI in patients with high bleeding risk: A subgroup analysis from STOPDAPT-3. Circ. Cardiovasc. Interv..

[23] Guimarães P.O., Franken M., Tavares C.A.M., Antunes M.O., Silveira F.S., Andrade P.B., Bergo R.R., Joaquim R.M., de Paula J.E.T., Nascimento B.R. (2025). Early withdrawal of aspirin after PCI in acute coronary syndromes. N. Engl. J. Med..

[24] Tavares C.A.M., Guimarães P.O., Franken M., Junior J.M., Lemos S., Almeida B.O., Martins E.B., Figueiredo E.L., da Rocha A.M., Soares A.A. (2026). Potent P2Y12 inhibitor monotherapy vs. DAPT after PCI in patients with and without STEMI: The NEO-MINDSET substudy. J. Am. Coll. Cardiol..

[25] Hong S.J., Lee S.J., Suh Y., Yun K.H., Kang T.S., Shin S., Kwon S.W., Lee J.W., Cho D.K., Park J.K. (2024). T-PASS (Ticagrelor Monotherapy in Patients Treated With New-Generation Drug-Eluting Stents for Acute Coronary Syndrome) Investigators. Stopping aspirin within 1 month after stenting for ticagrelor monotherapy in acute coronary syndrome: The T-PASS randomized noninferiority trial. Circulation.

[26] Ge Z., Kan J., Gao X., Raza A., Zhang J.J., Mohydin B.S., Gao F., Shao Y., Wang Y., Zeng H. (2024). Ticagrelor alone versus ticagrelor plus aspirin from month 1 to month 12 after PCI in patients with acute coronary syndromes (ULTIMATE-DAPT). Lancet.

[27] Vranckx P., Valgimigli M., Jüni P., Hamm C., Steg P.G., Heg D., van Es G.A., McFadden E.P., Onuma Y., van Meijeren C. (2018). Ticagrelor plus aspirin for 1 month, followed by ticagrelor monotherapy for 23 months vs aspirin plus clopidogrel or ticagrelor for 12 months after drug-eluting stent implantation (GLOBAL LEADERS). Lancet.

[28] Tarantini G., Honton B., Paradies V., Lemesle G., Range G., Godin M., Mangin L., Cuisset T., Ruiz-Nodar J.M., Brugaletta S. (2025). Early discontinuation of aspirin after PCI in low-risk acute myocardial infarction. N. Engl. J. Med..

[29] Lee Y.J., Shin S., Kwon S.W., Suh Y., Yun K.H., Kang T.S., Lee J.W., Cho D.K., Park J.K., Bae J.W. (2024). Ticagrelor monotherapy for acute coronary syndrome: An individual patient data meta-analysis of TICO and T-PASS trials. Eur. Heart J..

[30] Mehran R., Baber U., Sharma S.K., Cohen D.J., Angiolillo D.J., Briguori C., Cha J.Y., Collier T., Dangas G., Dudek D. (2019). Ticagrelor with or without aspirin in high-risk patients after PCI. N. Engl. J. Med..

[31] Kim B.K., Hong S.J., Cho Y.H., Yun K.H., Kim Y.H., Suh Y., Cho J.Y., Her A.Y., Cho S., Jeon D.W. (2020). Effect of ticagrelor monotherapy vs ticagrelor with aspirin on major bleeding and cardiovascular events in patients with acute coronary syndrome: The TICO randomized clinical trial. JAMA.

[32] Newby D.E., on behalf of the DUAL-ACS Investigators Duration of dual antiplatelet therapy after acute myocardial infarction: The DUAL-ACS randomised clinical trial. Proceedings of the ESC Congress 2025.

[33] Valgimigli M., Frigoli E., Heg D., Tijssen J., Jüni P., Vranckx P., Ozaki Y., Morice M.-C., Chevalier B., Onuma Y. (2021). Dual antiplatelet therapy after PCI in patients at high bleeding risk. N. Engl. J. Med..

[34] Hahn J.Y., Song Y.B., Oh J.H., Chun W.J., Park Y.H., Jang W.J., Im E.S., Jeong J.O., Cho B.R., Oh S.K. (2019). Effect of P2Y12 inhibitor monotherapy vs dual antiplatelet therapy on cardiovascular events in patients undergoing percutaneous coronary intervention: The SMART-CHOICE randomized clinical trial. JAMA.

[35] Watanabe H., Domei T., Morimoto T., Natsuaki M., Shiomi H., Toyota T., Ohya M., Suwa S., Takagi K., Nanasato M. (2019). Effect of 1-month dual antiplatelet therapy followed by clopidogrel vs 12-month dual antiplatelet therapy on cardiovascular and bleeding events in patients receiving PCI: The STOPDAPT-2 randomized clinical trial. JAMA.

[36] Watanabe H., Morimoto T., Natsuaki M., Yamamoto K., Obayashi Y., Ogita M., Suwa S., Isawa T., Domei T., Yamaji K. (2022). Comparison of clopidogrel monotherapy after 1 to 2 months of dual antiplatelet therapy with 12 months of dual antiplatelet therapy in patients with acute coronary syndrome: The STOPDAPT-2 ACS randomized clinical trial. JAMA Cardiol..

[37] Watanabe H., Morimoto T., Natsuaki M., Yamamoto K., Obayashi Y., Nishikawa R., Kimura T., Imada K., Suwa S., Isawa T. (2026). Clopidogrel versus aspirin monotherapy beyond 1 year after PCI: The final 5-year results of the STOPDAPT-2 ACS and STOPDAPT-2 total cohort. Circ. Cardiovasc. Interv..

[38] Valgimigli M., Hong S.-J., Gragnano F., Chalkou K., Franzone A., da Costa B.R., Baber U., Kim B.-K., Jang Y., Chen S.-L. (2024). De-escalation to ticagrelor monotherapy versus 12 months of dual antiplatelet therapy in patients with and without acute coronary syndromes: A systematic review and individual patient-level meta-analysis of randomised trials. Lancet.

[39] Claassens D.M.F., Vos G.J.A., Bergmeijer T.O., Hermanides R.S., Van’t Hof A.W., Van Der Harst P., Barbato E., Morisco C., Gin R.M.T.J., Asselbergs F.W. (2019). A genotype-guided strategy for oral P2Y12 inhibitors in primary PCI. N. Engl. J. Med..

[40] Jang Y., Park S.D., Lee J.P., Choi S.H., Kong M.G., Won Y.S., Kim M., Lee K.H., Han S.H., Kwon S.W. (2025). One-month dual antiplatelet therapy followed by prasugrel monotherapy at a reduced dose: The 4D-ACS randomised trial. EuroIntervention.

[41] Kim H.S., Kang J., Hwang D., Han J.-K., Yang H.-M., Kang H.-J., Koo B.-K., Rhew J.Y., Chun K.-J., Lim Y.-H. (2020). Prasugrel-based de-escalation of dual antiplatelet therapy after percutaneous coronary intervention in patients with acute coronary syndrome (HOST-REDUCE-POLYTECH-ACS): An open-label, multicentre, non-inferiority randomised trial. Lancet.

[42] Li Y., Li J., Wang B., Jing Q., Zeng Y., Hou A., Wang Z., Liu A., Zhang J., Zhang Y. (2024). Extended clopidogrel monotherapy vs DAPT in patients with acute coronary syndromes at high ischemic and bleeding risk: The OPT-BIRISK randomized clinical trial. JAMA Cardiol..

[43] Sibbing D., Aradi D., Jacobshagen C., Gross L., Trenk D., Geisler T., Orban M., Hadamitzky M., Merkely B., Kiss R.G. (2017). Guided de-escalation of antiplatelet treatment in patients with acute coronary syndrome undergoing percutaneous coronary intervention (TROPICAL-ACS). Lancet.

[44] Vrints C., Andreotti F., Koskinas K.C., Rossello X., Adamo M., Ainslie J., Banning A.P., Budaj A., Buechel R.R., Chiariello G.A. (2024). 2024 ESC Guidelines for the management of chronic coronary syndromes. Eur. Heart J..

[45] Rao S.V., O’Donoghue M.L., Ruel M., Rab T., Tamis-Holland J.E., Alexander J.H., Baber U., Baker H., Cohen M.G., Cruz-Ruiz M. (2025). 2025 ACC/AHA/ACEP/NAEMSP/SCAI guideline for the management of patients with acute coronary syndromes. Circulation.

[46] Costa F., van Klaveren D., James S., Heg D., Räber L., Feres F., Pilgrim T., Hong M.-K., Kim H.-S., Colombo A. (2017). Derivation and validation of the predicting bleeding complications in patients undergoing stent implantation and subsequent dual antiplatelet therapy (PRECISE-DAPT) score. Lancet.

[47] Yeh R.W., Secemsky E.A., Kereiakes D.J., Normand S.-L.T., Gershlick A.H., Cohen D.J., Spertus J.A., Steg P.G., Cutlip D.E., Rinaldi M.J. (2016). Development and validation of a prediction rule for benefit and harm of dual antiplatelet therapy beyond 1 year after percutaneous coronary intervention. JAMA.

[48] Gragnano F., van Klaveren D., Heg D., Räber L., Krucoff M.W., Raposeiras-Roubín S., Berg J.M.T., Leonardi S., Kimura T., Corpataux N. (2025). Derivation and validation of the PRECISE-HBR score to predict bleeding after percutaneous coronary intervention. Circulation.

[49] Capodanno D., Angiolillo D.J. (2023). Personalised antiplatelet therapies for coronary artery disease: What the future holds. Eur. Heart J..

[50] Gibson C.M., Mehran R., Bode C., Halperin J., Verheugt F.W., Wildgoose P., Birmingham M., Ianus J., Burton P., van Eickels M. (2016). Prevention of bleeding in patients with atrial fibrillation undergoing PCI. N. Engl. J. Med..

[51] Cannon C.P., Bhatt D.L., Oldgren J., Lip G.Y., Ellis S.G., Kimura T., Maeng M., Merkely B., Zeymer U., Gropper S. (2017). Dual antithrombotic therapy with dabigatran after PCI in atrial fibrillation. N. Engl. J. Med..

[52] Lopes R.D., Heizer G., Aronson R., Vora A.N., Massaro T., Mehran R., Goodman S.G., Windecker S., Darius H., Li J. (2019). Antithrombotic therapy after acute coronary syndrome or PCI in atrial fibrillation. N. Engl. J. Med..

[53] Vranckx P., Valgimigli M., Eckardt L., Tijssen J., Lewalter T., Gargiulo G., Batushkin V., Campo G., Lysak Z., Vakaliuk I. (2019). Edoxaban-based versus vitamin K antagonist-based antithrombotic regimen after successful coronary stenting in patients with atrial fibrillation (ENTRUST-AF PCI). Lancet.

[54] Hillis L.D., Smith P.K., Anderson J.L., Bittl J.A., Bridges C.R., Byrne J.G., Cigarroa J.E., Disesa V.J., Hiratzka L.F., Hutter A.M. (2011). 2011 ACCF/AHA guideline for coronary artery bypass graft surgery: Executive summary. Circulation.

[55] Yuan X., on behalf of the TOP-CABG Investigators De-escalated versus standard dual antiplatelet therapy after coronary artery bypass grafting: The TOP-CABG randomised trial. Proceedings of the ESC Congress 2025.

[56] Jeppsson A., James S., Möller C.H., Malm C.J., Dalén M., Vanky F., Modrau I.S., Andersen K., Anttila V., Atroshchenko G.V. (2025). Ticagrelor and aspirin or aspirin alone after coronary surgery for acute coronary syndrome: The TACSI randomised trial. N. Engl. J. Med..

[57] Burger W., Chemnitius J.M., Kneissl G.D., Rücker G. (2005). Low-dose aspirin for secondary cardiovascular prevention–cardiovascular risks after its perioperative withdrawal versus bleeding risks with its continuation–review and meta-analysis. J. Intern. Med..

[58] Gansevoort R.T., Correa-Rotter R., Hemmelgarn B.R., Jafar T.H., Heerspink H.J.L., Mann J.F., Matsushita K., Wen C.P. (2013). Chronic kidney disease and cardiovascular risk. Lancet.

[59] Graham C.A., Tan M.K., Chew D.P., Gale C.P., Fox K.A.A., Bagai A., Henderson M.A., Quraishi A.U.R., Déry J.-P., Cheema A.N. (2022). Use and outcomes of dual antiplatelet therapy for acute coronary syndrome in patients with chronic kidney disease. Heart Vessel..

[60] Gargiulo G., Windecker S., da Costa B.R., Feres F., Hong M.-K., Gilard M., Kim H.-S., Colombo A., Bhatt D.L., Kim B.-K. (2016). Short term versus long term dual antiplatelet therapy after implantation of drug eluting stent in patients with or without diabetes. BMJ.

[61] Joseph M., Krishna M.M., Ezenna C., Pereira V., Ismayl M., Nanna M.G., Bangalore S., Goldsweig A.M. (2025). Short versus one-year dual antiplatelet therapy after percutaneous coronary intervention: An updated meta-analysis. Am. J. Cardiol..

[62] Gragnano F., Cao D., Pirondini L., Franzone A., Kim H.-S., von Scheidt M., Pettersen A.R., Zhao Q., Woodward M., Chiarito M. (2023). P2Y12 inhibitor or aspirin monotherapy for secondary prevention of coronary events. J. Am. Coll. Cardiol..

[63] Kang J., Park K.W., Lee H., Hwang D., Yang H.-M., Rha S.-W., Bae J.-W., Lee N.H., Hur S.-H., Han J.-K. (2023). Aspirin versus clopidogrel for long-term maintenance monotherapy after percutaneous coronary intervention: The HOST-EXAM Extended study. Circulation.

[64] Giacoppo D., Gragnano F., Watanabe H., Kimura T., Kang J., Park K.-W., Kim H.-S., Pettersen A., Bhatt D.L., Pocock S. (2025). P2Y12 inhibitor or aspirin after percutaneous coronary intervention: Individual patient data meta-analysis of randomised clinical trials. BMJ.

[65] Valgimigli M., Choi K.H., Giacoppo D., Gragnano F., Kimura T., Watanabe H., Kim H.-S., Kang J., Park K.W., Pettersen A. (2025). Clopidogrel versus aspirin for secondary prevention of coronary artery disease: A systematic review and individual patient data meta-analysis. Lancet.

[66] Kang J., Park S., Yang H.M., Shin E.S., Rha S.W., Bae J.W., Lee N.H., Yoon H.J., Cho Y.H., Kim U. (2026). Aspirin versus clopidogrel for chronic maintenance monotherapy after percutaneous coronary intervention: 10-year follow-up of the HOST-EXAM trial. Lancet.

[67] Laudani C., Giacoppo D., Occhipinti G., Galli M., Greco A., Spagnolo M., Ortega-Paz L., Costa F., Angiolillo D.J., Capodanno D. (2025). Aspirin or P2Y12 inhibitor monotherapy after percutaneous coronary intervention for acute coronary syndromes. JACC Cardiovasc. Interv..

[68] Bonaca M.P., Bhatt D.L., Cohen M., Steg P.G., Storey R.F., Jensen E.C., Magnani G., Bansilal S., Fish M.P., Im K. (2015). Long-term use of ticagrelor in patients with prior myocardial infarction. N. Engl. J. Med..

[69] Storey R.F., Gurbel P.A., Ten Berg J., Bernaud C., Dangas G.D., Frenoux J.M., Gorog D.A., Hmissi A., Kunadian V., James S.K. (2020). Pharmacodynamics, pharmacokinetics, and safety of single-dose subcutaneous administration of selatogrel, a novel P2Y12 receptor antagonist, in patients with chronic coronary syndromes. Eur. Heart J..

[70] Nash J., Meah M.N., Whittington B., Debono S., Raftis J., Miller M.R., Sorbie A., Mills N.L., Nespoux J., Bruce L. (2024). PAR4 antagonism in patients with coronary artery disease receiving antiplatelet therapies. Arterioscler. Thromb. Vasc. Biol..

[71] Galli M., Laudani C., Occhipinti G., Spagnolo M., Gragnano F., D’Amario D., Navarese E.P., Mehran R., Valgimigli M., Capodanno D. (2024). P2Y12 inhibitor monotherapy after short DAPT in acute coronary syndrome: A systematic review and meta-analysis. Eur. Heart J. Cardiovasc. Pharmacother..

[72] Holm N.R., Andreasen L.D., Neghabat O., Laanmets P., Kumsars I., Bennett J., Olsen N.T., Odenstedt J., Hoffmann P., Dens J. (2023). OCT or angiography guidance for PCI in complex bifurcation lesions. N. Engl. J. Med..

[73] Ali Z.A., Landmesser U., Maehara A., Matsumura M., Shlofmitz R.A., Guagliumi G., Price M.J., Hill J.M., Akasaka T., Prati F. (2023). Optical coherence tomography-guided versus angiography-guided PCI (ILUMIEN IV). N. Engl. J. Med..

